# Use of the Smartphone and Self-Concept in University Students According to the Gender Variable

**DOI:** 10.3390/ijerph17124184

**Published:** 2020-06-12

**Authors:** Javier Cachón-Zagalaz, Déborah Sanabrias-Moreno, María Sánchez-Zafra, María Luisa Zagalaz-Sánchez, Amador Jesús Lara-Sánchez

**Affiliations:** Department of Didactics of Musical, Plastic and Corporal Expression, University of Jaén, 23071 Jaén, Spain; jcachon@ujaen.es (J.C.-Z.); dsmoreno@ujaen.es (D.S.-M.); lzagalaz@ujaen.es (M.L.Z.-S.); alara@ujaen.es (A.J.L.-S.)

**Keywords:** self-concept, smartphone, university, relationship, psychosocial factors

## Abstract

The university stage comprises a very important and vital period in the modification of students’ lifestyles, and these changes can affect their self-concept. The excessive use of technology today can also influence the formation of their identity. The aim of this study is to analyze the relationship between self-concept and the use of the smartphone by university students in terms of gender. The sample included 253 students (mean age 21.39 ± 3.27) of the Primary Education Degree of the University of Jaén (106 men and 147 women). A questionnaire was used to unify several instruments: a sociodemographic questionnaire, a Self-Concept Form-5 questionnaire (AF-5), and a questionnaire on cell phone-related experiences (CERM). The results show the existence of significant differences between both genders in the academic, emotional, and social dimensions of self-concept, with women showing a greater academic self-concept and men showing an emotional and physical one. Regarding the use of the smartphone in relation to self-concept, significant differences are found in the academic and emotional dimensions depending on the degree of use. In addition, in relation to the use of the smartphone, it has been detected that half of the students present potential problems. It is concluded that there is a relationship between both constructs, especially the academic and emotional self-concept.

## 1. Introduction

The university stage coincides with what some authors describe as emerging adulthood, a period of life in which people between the ages of 18 and 29 have passed through adolescence but have not yet fully assumed the role of an adult [1,2,3,4,5,6]. This cycle also coincides with a total change in lifestyle, since it involves interaction with a new group of people, the abandonment of the family nucleus in many cases, and the possible combination of studies and work. It is a time that can cause certain instability, since it is the moment when the subject strengthens his personality [7].

Related to these cognitive and psychological processes arises the term self-concept, a construct of high importance in this stage of change and consolidation of personal identity which is defined as the perception that an individual has of himself, taking into account limitations, characteristics, and personal relationships [8,9,10]. This self-perception is flexible and modifiable—that is, it is not the same throughout an individual’s life and is influenced by all the changes that the subject experiences in the different areas of his or her day-to-day life and furthermore, is influenced by the reinforcements, positive or negative, that other people exert on the individual [11]. Some authors argue that there are differences in some dimensions of self-concept according to gender, as indicated, for example, in the study by Cachón et al. (2015) [8].

The concept has also undergone several changes in its definition over the last few decades. In the seventies of the last century, the self-concept had a one-dimensional character—it was a single factor. In this line, the Rosenberg Self-esteem Scale [12], which measured this single dimension, became popular. Over the years, the deepening of the study of this construct led the scientific community to consider its multidimensional nature. In this sense, the study by Shavelson et al. (1976) [13] already defined self-concept as a hierarchical model formed by several factors or dimensions, so that a person can have a high self-concept in several aspects of his life and a low one in others.

Currently, the model that divides the self-concept into five dimensions is formally accepted: physical, related to body image and physical factors; emotional, understood as the ability to manage emotions that the individual has; family and perception about the role within the family; academic, in his role as a student; and social, conditioned by the quantity and quality of social relationships [14,15,16,17]. The Self-Concept Form-5 questionnaire by García and Musitu (1999) [18] is the most widely used in this regard.

On the other hand, it cannot be ignored that today’s society is technological and constantly changing. Technological advances have a great impact on people’s lives, on the activities they carry out, and on their socialization [19]. Mobile phones or smartphones (terms that will be used interchangeably in this paper) are currently one of the most famous products, are within the reach of almost all subjects, and are part of their lives. The use of these devices has become widespread, and it is common to manipulate them at any time of day, walking on the street, on public transport, at home, or even in class or at work.

According to the Ditrendia report [20], which analyses various aspects of mobile phone use, there were more than five billion users in 2019. In the specific case of Spain, 96% of the population uses them to access the Internet. In 2018, it has been estimated that users worldwide spent an average of 800 h using their smartphones to surf the Internet, and this figure is expected to increase to 930 h in 2021.

The COVID-19 pandemic has broken these forecasts, with mobile phone use skyrocketing in 2020 during periods of confinement. For example, in Spain during the second week of March and coinciding with the beginning of the state of alarm, mobile phone use increased by 38.3% compared to the last week of February. In general, the use of communication applications has increased by more than 50%, social networks by 20.9%, and television and cinema by a similar percentage. Among the most used applications in the communication sector are “Hangouts”; “Whatsapp”; and “Calls”, especially video calls, while in the social network sector are “Twitter”, “Facebook”, and “Instagram”. The television and film applications that have increased their use the most are “Megadede”, “Netflix”, and “Prime Video” [21].

Returning to the use of mobile phones, 85% of Spanish users use messaging applications, with WhatsApp being the most widely used, especially among young people between 14 and 24 years old. The second most applied activity by the Spanish is related to the display of videos (82%), and the third is the display of mobile mapping programs (75%). The favorite applications are games, social networks, entertainment, and photography [20].

As we have maintained, the smartphone is now a must-have for many people. Throughout the history of mankind, there are few elements that have been so influential in the lives of human beings, so the problem of dependence that it manages to create in its users is generated, and this can even affect relationships with other people. It is at this point that we talk about social problems related to the use of mobile phones [22], which are complemented by others that are also derived from this excessive use, such as lack of sleep, loss of the notion of time, obsession with what is happening on social networks, or the failure to do other important activities. Despite the fact that this device is used by both genders, some authors report that women spend the most time using it [23].

The use of the smartphone is especially noticeable among young people. The group of university students, along with teenagers, is one of the most likely to suffer from addiction problems, since the mobile phone is a first and indispensable object for them, and they consider it a fundamental tool to relate to and socialize with others [24]. The media is an essential tool for young people, but at this age its use is dangerous as it can influence people and change their behavior without them being aware [25]. The excessive use of the smartphone can cause health problems affecting sleep, increase the risk of having a traffic accident or influence academic results. In relation to this term, the concept of “phubbing” arises, understood as the fact of neglecting other people by continuously using the smartphone [26].

It should be noted that the two main constructs of this research (self-concept and the use of the smartphone) can influence each other, since one of the transcendental uses that young people give to the mobile phone is access to social networks in which they continually interact by publishing photographs with the intention of showing a lifestyle, real or imaginary. This digital individual is changeable and usually responds to the desires of the real subject, to how he wants to be seen or how he wants to see himself. It is essential to educate students about the dangers that can be caused by social networks, reminding them that the information they see published is usually subject to filters that transmit unreal information or with a very biased truth [27]

However, this process may not always be negative; the problem arises when the real self is lost and an identity crisis appears, influencing its self-concept [28]. Especially in the adolescent stage, it is very important to acquire and maintain a good body image, since this is directly related to the person’s general health [29]. Likewise, studies such as Pedrero et al. (2012) [30] state that the excessive use of mobile phones can make it even more difficult for people with a low self-concept to socialize openly and directly or even to speak of depressive symptoms.

Some people with low self-esteem and self-concept have trouble interacting with other subjects face-to-face, causing them to prefer to communicate with others through the smartphone, which makes them feel more confident. Therefore, people with this low self-concept may have an excessive use of the smartphone [31]. Twenge et al. (2018) [32] also comment that teenagers who spend less time using technological devices are happier than those who use them continuously.

Based on the information found, the hypothesis is that mobile phone use directly affects the self-concept of university students. Therefore, the main objective of this study is to analyze the relationship between the self-concept of university students and the use they make of their mobile phones, also analyzing the gender variable.

## 2. Materials and Methods

### 2.1. Participants

The sample is made up of 253 university students (*n* = 253) of the Primary Education Degree of the University of Jaén (Spain). A non-probabilistic sampling of accidental or casual type has been used. The distribution of participants according to gender is as follows: 106 men (41.9%) and 147 women (58.1%). The mean age of the sample subjects is 21.39 (±3.27); the minimum age of the participants is 18 and the maximum is 42 (range = 24 years).

### 2.2. Instruments

- Socio-demographic questionnaire (Ah-hoc): To analyze the gender of the subjects surveyed.
- Self-Concept Form-5 questionnaire (AF-5) by García and Musitu (1999) [18]: It consists of 30 items differentiated in 5 dimensions: academic (1, 6, 11, 16, 21 and 26), social (2, 7, 12, 17, 22 and 27), emotional (3, 8, 13, 18, 23 and 28), family (4, 9, 14, 19, 24 and 29), and physical (5, 10, 15, 20, 25 and 30). An example of an item is: “It’s difficult for me to make friends”. The type of response is a Likert scale of between 1 and 5 points, with 1 being “Never” and 5 being “Always”. In the participants of the study, the initial reliability (including all items) of the scores obtained (Cronbach’s alpha) was 0.64 for the total of the scale, for the academic dimension 0.81, for the social dimension 0.79, for the emotional dimension 0.65, for the family dimension 0.86, and for the physical dimension 0.78. In the study, item number 8, or “Many things make me nervous”, has been eliminated because it interfered with the reliability of the scale. Excluding this item from the analyses, the total reliability of the scale was 0.87.
- Questionnaire on cell phone-related experiences (CERM—Cuestionario de Experiencias Relacionadas con el Móvil), by Beranuy et al. (2009) [33]: Scale made up of 10 items answered on a four-point Likert scale, with 1 being “Almost never” and 4 being “Almost always”. An example of an item is “Do you get angry or irritated when someone bothers you while using your mobile phone?”. Following authors such as Carbonell et al. (2012) [34], the results have been analyzed by grouping the participants into three groups: “No problems” (10 to 15 points), “Occasional problems” (16 to 23 points), and “Severe problems” (24 to 40 points). The reliability of the scale (Cronbach’s alpha) was 0.75.

### 2.3. Procedure

Firstly, a questionnaire was developed by linking the above instruments and applying them collectively and voluntarily to participants in their classrooms. At the beginning, they were informed of the nature of the study and were assured of the anonymity of the responses and results.

This research also meets the international ethical standards set by the World Medical Association (WMA), which issued the Declaration of Helsinki in 1964 [35], although it has undergone subsequent revisions, the latest in 2017. It also complies with the Spanish legislation required for this type of work.

### 2.4. Data Analysis

The SPSS 22.0 (IBM corps., Armonk, NY, USA) statistical program was used for data analysis. A descriptive study was performed to report the characteristics of the sample subjects (means and standard deviations) and various non-parametric tests (Spearman’s rho correlation, Mann–Whitney U test, H Kruskall Wallis), since the assumption of normality was based on the results obtained in the Kolmogorov–Smirnov test (*n* > 30) was not met.

## 3. Results

### 3.1. Statistical Tests Related to Self-Concept

Table 1 shows the results when bivariate correlations are made between the different dimensions of the self-concept, as well as the mean (M) and standard deviation of each one of them. The highest dimension presented by university students is the family dimension (M = 26.06, ±3.89), followed by the social dimension (M = 23.10, ±3.66) and the academic dimension (M = 21.80, ±3.29). The emotional self-concept is the one with the lowest mean (M = 15.47, ±3.05), followed by the physical one (M = 20.31, ±4.13). Regarding correlations, the academic self-concept correlates significantly and positively with the social dimension (*rho*(253) = 0.259, *p* = 0.000), the family dimension (*rho*(253) = 0.269, *p* = 0.000), and the physical dimension (*rho*(253) = 0.288, *p* = 0.000). The social self-concept significantly and positively correlates with the emotional (*rho*(253) = 0.270, *p* = 0.000), familial (*rho*(253) = 0.357, *p* = 0.000), and physical (*rho*(253) = 0.441, *p* = 0.000) self-concepts. The emotional dimension correlates positively and significantly with the familial (*rho*(253) = 0.154, *p* = 0.014) and physical (*rho*(253) = 0.357, *p* = 0.000) dimensions. Finally, the family self-concept correlates positively and significantly with the physical self-concept (*rho*(253) = 0.242, *p* = 0.000).

When the U Mann–Whitney test was performed to relate the dimensions of self-concept with the gender variable (Table 2), statistically significant differences (*p* < 0.01) were obtained in favor of girls in academic self-concept (*Z* = −3.286, *p* = 0.001, r = 0.020) and of boys in emotional (*Z* = −5.456, *p* = 0.000, r = 0.35) and physical self-concepts (*Z* = −5.640, *p* = 0.000, r = 0.34). In relation to the size of the effect, the values obtained in the variables in which significant differences have been found are of medium size (*r* < 0.50, *r* > 0.30). It can be observed that those dimensions with statistically significant differences according to gender have a higher statistical power.

### 3.2. Analysis of Smartphone Use

As can be seen in Table 3, most university students are classified between those who do not have problems related to mobile phone use (*n* = 102, 40.3%) and those who may have them (*n* = 142, 56.1%). Only nine students present severe problems (3.6%). In terms of gender differentiation, girls show less problems than boys (*n* = 59 vs. *n* = 43), but at the same time they are the ones that show more potential difficulties (*n* = 84 vs. *n* = 58). In terms of severe problems, both genders are equal, with the male being slightly superior (*n* = 5 vs *n* = 4).

### 3.3. Relationship between the Dimensions of the Self-Concept and the Use of the Smartphone

Table 4 shows the relationship between the dimensions of self-concept and the three categories of mobile phone use. Statistically significant differences (*p* < *0*.05) were obtained in the academic dimension (χ^2^ = 7.003, *p* = *0*.030, r = 0.027) and in the emotional dimension (χ^2^ = 17.351, *p* = 0.000, r = 0.082), being in both the mean of the category “No problems” the highest (M = 22.38, SD = 3.44 vs. M = 16.47, SD = 2.87). It is observed that the statistical power is higher in those variables in which there are statistically significant differences.

## 4. Discussion

The results obtained show that of the five dimensions into which the self-concept is divided, the one that scores most is the family one, followed by the social one. These data agree with Bustos et al. (2015) [36] and Baptista et al. (2012) [14], for whom the family relationship is a key factor in the lives of people that can affect, positively or negatively, other key aspects of the lives of individuals. This is even more important at the university stage, because most students leave the family nucleus and losing family closeness can affect other aspects of their lives. Therefore, it is a very positive fact that the subjects surveyed show high scores in this dimension.

With regard to social self-concept, it is important to remember that university students create social bonds with people they meet during their years of study at this institution. A high social self-concept implies, in most cases, that they have no problems in creating new relationships with their peers [37].

The order in which these dimensions are scored continues with the physical and academic dimensions, with the emotional one being the one with the lowest score. Logically, the physical self-concept is closely related to physical activity practice, so one way to increase it would be to perform physical exercise regularly [17,38]. Some authors indicate that academic self-concept is influenced by some variables, such as student involvement in the teaching process or the relationship between the teacher and student [9]. It is perhaps for this reason that university students score lower on the academic dimension than on others, given that the relationship between students and teachers at the university is not as close as it is at other educational stages. Finally, as for the emotional dimension, which is the one that presents the lowest scores, it has been found that it is closely related to the social one. This is due to the fact that people who consider themselves emotionally strong have more facility when it comes to strengthening ties with other subjects [39].

According to the results, there are statistically significant differences (*p* < *0*.01) between both genders in the academic, emotional, and physical dimensions. Women present a higher academic self-concept than men, possibly because they get better grades than men by having better study habits [40]. In contrast to this idea, the article by Jiménez-Caballero et al. (2015) [41] concludes that gender is not a variable that influences the academic performance of university students. With regard to the emotional and physical dimension, it is men who have higher scores, data which is consistent with Chacón-Cuberos et al. (2020) [7]. Suárez and Wilches (2015) [42] claim that men perceive and regulate their emotions better than women. It is important to pay attention to your emotions, because not doing so can lead to some symptoms of depression. In terms of the physical self-concept, Linares-Manrique et al. (2016) [43] states that men have a superior appreciation of themselves in terms of physical ability, physical condition, strength, and physical attractiveness.

As for the use of mobile devices, more than 50% of the sample presents potential problems. It should not be forgotten that most of the university students surveyed belong to what is known as the digital native generation, understood as the people who were born when they switched from the analog to the digital world [19].

In terms of gender differentiation, girls are the group that stands out in the “no problem” category, although they outnumber boys with potential complications related to their use. These results coincide with those of López-Rosales and Jasso-Medrano (2019) [23], who found differences between the time of use of mobile phones between both genders but not addictive behaviors. According to these authors, girls use social networks more frequently to express themselves through them and boys to meet other people. Zagalaz et al. (2019) [44] also state that women use their smartphones more, especially to access social networks, and that men prefer to use them to surf the Internet or play video games.

When analyzing the results obtained in the relationship between the dimensions of self-concept and the use of the smartphone, it is found that there are statistically significant differences (*p* < 0.05) in the academic and emotional self-concepts, with those subjects who have severe problems with the use of the mobile phone scoring in both dimensions lower. The students are aware that the excessive use of smartphones can reduce their university performance and, as a consequence, their academic self-concept. According to the data obtained by Herrera et al. (2014) [45], more than 50% of the young participants in their research stated that they were distracted in class when using their mobile phone.

In terms of emotional self-concept, the results are reinforced by Olivencia-Carrión et al. (2016) [22], who state that the use of mobile devices can create situations of dependency and emotional attachment, and that they are used to deal with negative emotional situations, such as boredom.

## 5. Conclusions

Finally, as the main conclusion, it should be noted that university students show a high degree of family self-concept, followed by the social and physical self-concepts. The academic and emotional dimensions score the lowest. As for the differentiation by gender, this are found only in the academic self-concept in favor of women and in the emotional and physical dimensions in favor of men.

Regarding the use of smartphones, more than half of the students surveyed present potential problems related to their use, with a small percentage having severe problems. Finally, the relationship between self-concept and mobile phone use is remarkable in the academic and emotional dimensions, being the only ones in which significant differences appear depending on the degree of use of smartphones.

## Figures and Tables

**Table 1:** Spearman’s bivariate correlations and descriptive statistics of the self-concept.

Self-Concept Type	Academic Self-Concept	Social Self-Concept	Emotional Self-Concept	Family Self-Concept	Physical Self-Concept
Academic self-concept	-	0.259 **	0.092	0.269 **	0.288 **
Social self-concept	0.259 **	-	0.270 **	0.357 **	0.441 **
Emotional self-concept	0.092	0.270 **	-	0.154 *	0.357 **
Family self-concept	0.269 **	0.357 **	0.154 *	-	0.242 **
Physical self-concept	0.288 **	0.441 **	0.357 **	0.242 **	-
M (SD)	21.80 (±3.29)	23.10 (±3.66)	15.47 (±3.05)	26.06 (±3.89)	20.31 (±4.13)
*α*	0.81	0.79	0.65	0.86	0.78

M, mean; SD, standard deviation. * The correlation is significant at the 0.05 level (bilateral)., ** Correlation is significant at the 0.01 level.

**Table 2:** U Mann–Whitney test relating self-concept to gender.

Self-Concept Type	Men M (SD)	Women M (SD)	Total M (SD)	*Z*	*p*	*r*	1 *− β*
Academic self-concept	21.02 (±3.56)	22.36 (±2.97)	21.80 (±3.29)	−3.286	0.001 *	0.200	0.895
Social self-concept	23.60 (±3.73)	22.74 (±3.58)	23.10 (±3.66)	−1.683	0.092	0.116	0.450
Emotional self-concept	16.72 (±2.72)	14.57 (±2.96)	15.47 (±3.05)	−5.456	0.000 *	0.353	1
Family self-concept	25.75 (±4.08)	26.29 (±3.75)	26.06 (±3.89)	−1.175	0.240	0.068	0.191
Physical self-concept	21.98 (±4.04)	19.11 (±3.77)	20.31 (±4.13)	−5.640	0.000 *	0.344	1

* statistically significant differences at the 0.01 level.

**Table 3:** Descriptive statistics of mobile phone use by gender.

Gender	No Problems		Occasional Problems		Severe Problems	
	*n*	Total (%)	*n*	Total (%)	*n*	Total (%)
Male	43	102 (40.3%)	58	142 (56.1%)	5	9 (3.6%)
Female	59		84		4	

**Table 4:** H Kruskall Wallis test between self-concept and mobile use.

Self-Concept	Addiction Level	M (SD)	χ^2^	Sig.	*r*	1 − *β*
Academic self-concept	No problems	22.38 (3.44)	7.003	0.030 *	0.027	0.643
	Occasional problems	21.49 (3.16)				
	Severe problems	20.11 (2.66)				
Social self-concept	No problems	23.41 (3.82)	3.206	0.201	0.010	0.269
	Occasional problems	22.98 (3.44)				
	Severe problems	21.55 (4.95)				
Emotional self-concept	No problems	16.47 (2.87)	17.351	0.000 *	0.082	0.992
	Occasional problems	14.90 (13.22)				
	Severe problems	13.22 (3.30)				
Family self-concept	No problems	26.19 (4.65)	5.327	0.070	0.003	0.102
	Occasional problems	26.03 (3.21)				
	Severe problems	25.11 (4.53)				
Physical self-concept	No problems	20.50 (4.12)	1.822	0.402	0.003	0.104
	Occasional problems	20.23 (4.15)				
	Severe problems	19.44 (4.30)				

Sig., significance. * statistically significant differences at the 0.05 level.
